# β-Aminobutyric Acid Induced Resistance against *Alternaria* Fruit Rot in Apple Fruits

**DOI:** 10.3390/jof7070564

**Published:** 2021-07-14

**Authors:** Lior Gur, Moshe Reuveni, Yigal Cohen

**Affiliations:** 1 Shamir Research Institute, University of Haifa, Katzrin 1290000, Israel; liogur@gmail.com; 2 Faculty of Life Sciences, Bar-Ilan University, Ramat Gan 5290002, Israel; yigal.cohen1@gmail.com

**Keywords:** *Alternaria mali*, *Alternaria alternata* apple pathotype, BABA, disease control, induced resistance, plant defense activators, priming, integrated pest-management

## Abstract

Fruit body rot and calyx rot caused by *Alternaria alternata* f. sp. *mali* is an important disease of apple worldwide. The disease has recently become severe in cv. Pink Lady apple in Israel to an extent that has never been reported elsewhere in the world. No alternative control measures of the disease except fungicides are known. Here, we show for the first time that dl-β-aminobutyric acid (BABA) induces resistance against *Alternaria* fruit rot (AFR) in apple fruits in the laboratory and in the orchard. AFR was inhibited in fruits treated with BABA of 1000 μg/mL. BABA did not inhibit spore germination or mycelial growth of the pathogen in vitro (up to 2000 μg/mL). It was most inhibitory when applied 4 days prior to inoculation of detached fruits. BABA inhibited AFR also curatively when applied at 24 h post inoculation. Five other isomers of aminobutyric acid failed to protect the fruits from rot formation. Three field trials in commercial apple orchards proved that BABA was as protective against AFR as the commercial standard fungicidal mixture of azoxystrobin and difenoconazole. This research suggests that BABA may serve as a resistance inducer in apple against AFR. It can be used as an adequate alternative to the currently used fungicides or integrated in disease management programs to reduce fungicide load and buildup of fungicide resistance.

## 1. Introduction

*Alternaria* leaf blotch and fruit spot of apple (*Malus domestica* Borkh), caused by the fungus *Alternaria alternata* f. sp. *mali* (syn. *Alternaria mali* Roberts, or *Alternaria alternata* apple pathotype) [1,2,3], is an important disease of apple in many countries [4,5,6,7,8,9,10], including Israel [11]. The disease affects cultivars such as Golden Delicious, Starking Delicious, Gala and Pink Lady [5,8,11,12,13]. In most cases, fruit symptoms are usually limited to small, corky, dark lesions, often associated with the lenticels [12]. The pathogen may cause soft rot, particularly when the skin is wounded by mechanical damage or insects [14,15,16] or has cracks around the fruit calyx [17]. Severe outbreaks of apple fruit rot were observed in cv. Pink Lady in northern Israel [11]. Because large lesions and rots are the common symptoms in fruits in Israel, unlike the small spots seen in other countries, we named the disease ‘*Alternaria* fruit rot’ (AFR) [18]. Heavy infection with AFR is rare in other countries [16,19]. Lesions on the fruit body and adjacent to cracks around the calyx develop to form large, dark, rotted areas which can destroy 80% of the fruits in some orchards [11]. Whereas leaf infection and defoliation are the main damage caused by *A. alternata* f. sp. *mali* in most growing areas in the world [16,19], fruit rot is the major problem in Israel [11]. Pink Lady is the most valuable cultivar among apple cultivars grown in Israel due to its taste and flavor. Therefore, AFR is a major factor reducing apple fruit quality with a severe economic impact.

In previous studies, we showed that AFR may be controlled by foliar fungicidal sprays [20]. Integrated Pest Management (IPM) in the orchards encourages the use of alternative methods. The introduction of the plant defense activator dl-β-aminobutyric acid (BABA) by Cohen [21,22,23] provided a new option for chemical management of plant diseases based on the induction of host resistance.

BABA was shown to protect about 40 plant species against about 80 pests, including protista, bacteria, oomycetes, fungi, nematodes, arthropods and viruses. However, only a few studies showed efficacy of BABA against tree diseases [24,25,26,27,28].

The objective of the present study was to evaluate the inhibitory effect of BABA on *A. alternata* f. sp. *mali* in vitro, and on rot formation in apple fruit in the laboratory and the field. The data suggest that BABA induces protection against AFR in the laboratory and provides good control of apple rot in cv. Pink Lady in the orchard.

## 2. Materials and Methods

### 2.1. Chemicals

BABA (dl-3-amino-n-butanoic acid, dl-β-aminobutyric acid) was purchased from Sigma, Israel. AABA (α-aminobutyric acid), GABA (γ-aminobutyric acid), α-amino-iso-butyric acid, dl-β-iso BABA and 3-aminopropionic acid (β-alanine) were all purchased from Sigma-Aldrich (Rehovot, IL, Israel). These compounds were used for in vitro studies in the laboratory or with detached fruits. The efficacy of BABA was compared with that of the fungicide Score 250 SC (Syngenta, Basel, Switzerland), containing difenoconazole, a sterol demethylation inhibitor (DMI, FRAC Code 3) [29] and the commercial pre-mixed fungicide Ortiva-Top 325 SC (Syngenta, Basel, Switzerland) comprising azoxystrobin (FRAC Code 11) at 200 g/L and difenoconazole at 125 g/L. A wettable powder (25 WP) formulation of BABA (gratefully provided by Syngenta) was also used in field experiments. All concentrations in laboratory experiments are presented as μg/mL of active ingredient (a.i.), unless stated otherwise. The efficacy of BABA in controlling *Alternaria* fruit rot in apple fruits in the field was compared to that of the commercial pre-mixed fungicide Ortiva-Top 325 SC.

### 2.2. Pathogen

A single-spore culture of *A. alternata* f. sp. *mali* grown on potato dextrose agar (PDA 39 g/L, Difco (Detroit, MI, USA)) was maintained at 25 °C in 9 cm Petri dishes in the dark for 10–12 days until conidia were produced on developed colonies. Conidia were harvested from PDA plates by adding 3 mL of sterile distilled water to each plate and gently rubbing the sporulating mycelial mass with a bent glass rod. Spore concentration was adjusted with the aid of a hemocytometer to 5 × 10^5^ spores mL^−1^ [18]. A highly virulent isolate, PL-SH-B-3011, isolated from body rot of a Pink Lady fruit grown in Sha’al orchard (33°07′43″ N, 35°43′34″ E) in 2011, was used in all experiments [11].

### 2.3. In Vitro Assays

#### 2.3.1. Germination

Fresh conidial suspensions were kept in 2 mL tubes and mixed with an aqueous suspension of BABA to achieve a final concentration of 0, 10, 100, 1000 and 2000 μg/mL, or the pre-mixture azoxystrobin plus difenoconazole of 162.5 μg/mL (a.i) at a uniform conidial concentration of 2 × 10^4^ mL^−1^. A 70 μL aliquot of each mixture was pipetted into depressions of microscope slides (three per treatment) and incubated on a wet filter paper in 9 cm Petri dishes at 25 °C for 20 h in the dark. The number of germinating conidia was counted in each depression at ×100 magnification and percent germination determined. Each experiment was conducted twice.

#### 2.3.2. Mycelial Growth

Two 5 mm agar disks bearing *A. alternata* f. sp. *mali* were taken from the edge of a freshly growing colony and placed in a 9 cm petri dish containing PDA amended with 0, 10, 100, 1000 or 2000 μg/mL BABA, or 50 or 162.5 μg/mL (a.i) difenoconazole and the pre-mixture azoxystrobin plus difenoconazole, respectively. Cultures were incubated at 25 °C in the dark and colony diameter was recorded after 3, 4 and 5 days. Three petri dishes were used for each treatment concentration, and experiments were conducted twice.

#### 2.3.3. Growth in Liquid Medium

The activity of BABA, difenoconazole and the pre-mixture azoxystrobin plus difenoconazole on fungal growth was also examined in liquid medium. One milliliter of potato dextrose broth (PDB 24 g/L, Difco) containing the following compounds was added to sterile 48-well plates: BABA of 0, 10, 100, 1000 or 2000 μg/mL; difenoconazole of 25 μg/mL (a.i); and the pre-mixture azoxystrobin plus difenoconazole of 32.5 μg/mL (a/i). To each well, 4 μL of conidial suspension (2.5 × 10^4^ conidia mL^−1^, 100 conidia per well) was added. Five wells were used for each dose treatment. The plates were stirred (30 rpm) at 25 °C in the dark. Seven days after inoculation, the growing mycelial was taken out with the aid of tweezers, blotted dry on filter paper for 1 h in a chemical fume hood and weighed.

### 2.4. Efficacy in Detached Fruits

#### 2.4.1. Inoculation Procedure

Detached fruits were inoculated as described by Gur et al. [11]. Briefly, mature fruits, cv. Pink Lady from untreated trees, were brought from the orchard, washed with water, blotted dry with a paper towel, sprayed with 90% ethanol and allowed to dry at room temperature. Conidia were harvested from PDA cultures by adding 3 mL of sterile distilled water to each dish and gently rubbing the sporulating mycelial mats with a bent glass rod. The spore concentration was adjusted with the aid of a hemocytometer to 5 × 10^5^ spores/mL. Each fruit was wounded at six points on its surface with a 2 mm-diameter sterile plastic tip to a depth of ~3 mm and then inoculated by pipetting 10 μL conidial suspension to each wound. Following inoculation, fruits were placed on trays containing wet filter paper, covered with plastic bags to maintain high humidity and held covered in a growth chamber (25 °C) in the dark for 13 days.

#### 2.4.2. Experiment 1. Effect of Application Date on Rot Development

Aliquots of 300 μL of BABA (1000 μg/mL) were injected to each of six sites on each of three fruits per treatment, with the aid of an insulin injector, through the fruit skin into the mesoderm to a depth of ~12 mm. Injection was carried out at 0, 1, 2, 3 and 4 days before inoculation, or one day after inoculation. Injected sites were marked, and fruits were kept in the growth chamber at 25 °C until inoculated. Fruits injected with sterile water and fruits inoculated with conidial suspension in water served as controls.

#### 2.4.3. Experiment 2. Effect of BABA Concentration on Rot Development

BABA of 0, 10, 100, 500, 1000 and 2000 μg/mL were injected into fruits four days before inoculation as described above (300 μL per concentration; six sites on three fruits/treatment). Each injected site was marked, and fruits were kept in the growth chamber at 25 °C until inoculation was performed in each injected site as described above. Fruits injected with a mixture of BABA with conidial suspension, sterile water only or with conidial suspension in water served as controls.

#### 2.4.4. Experiment 3. Efficacy of Isomers of BABA

Aqueous solutions of BABA, AABA (α-aminobutyric acid), GABA (γ-aminobutyric acid), α-amino-iso-butyric acid, dl-β-iso-BABA or β-alanine, all at 1000 μg/mL, were each injected into fruits as described above, four days before inoculation, to each of six sites on each of three fruits per treatment. Fruits were inoculated and incubated as described above and 14 days after inoculation the rot diameter was recorded.

### 2.5. Efficacy in the Orchard

Three field experiments were conducted during August to October in commercial orchards of apple cv. Pink Lady in the Golan region in Israel. Fertilization, irrigation and other cultural practices in the orchards were as recommended by the Extension Service of the Ministry of Agriculture, Israel. The annual rainfall was 750–850 mm (Israel Meteorological Service, www.ims.gov.il, accessed on 2 March 2020), all of which occurs during the winter (October–April). During the spring (April–May), temperatures were moderate (10–25 °C), accompanied by leaf wetness due to rain events. In the summer (June–September) the average midday relative humidity (RH) and temperature were 35–40% and 30 °C, respectively. Skies were cloudless during most of the summer. Night temperature occasionally fell to 14–20 °C between May to August, and dew accumulated on leaf surfaces during some nights.

#### 2.5.1. Field Experiment 1 and 2

Two field experiments were conducted, in 2012 and 2020. BABA (dl-β-aminobutyric acid, 0.1% *w*/*v*) and the pre-mixture azoxystrobin plus difenoconazole (0.05% *w*/*v*) were tested in Ein Zivan orchard on cv. Pink Lady trees. In experiment 1, five shoots bearing 2 fruits each were selected and labeled on each of five different trees per treatment (10 fruits per treatment). In experiment 2, six shoots bearing 3 fruits each were selected and labeled on each of six different trees per treatment (18 fruit per treatment). Each fruit (mean diameter = 68 mm) was wounded with a 1.3 mm-diameter needle at four different sites. Branches including fruits were then sprayed using a handy sprayer with about 10 mL solution of each compound per branch. Shoots with non-treated fruits on untreated trees served as controls. Four days later, fruits were inoculated as described in Gur et al. [18]. Briefly, a conidial suspension of *A. alternata* f. sp. *mali* (5 × 10^5^ spores mL^−1^) was prepared and held on ice until fruits were spray-inoculated in the orchard. Following spray inoculation, each fruit was covered with a wet plastic bag to maintain high humidity. Bags were removed on the following morning. Thirteen days after inoculation, the diameter of fruit rot developed on the skin and mesocarp of each sprayed and inoculated fruit was measured.

#### 2.5.2. Field Experiment 3

BABA (formulated as 25 WP) (0.1% *w*/*v* a.i) and the pre-mixture azoxystrobin plus difenoconazole (0.05% *w*/*v*) were tested at Ein Zivan orchard on cv. Pink Lady trees in 2012. Five shoots containing 1–2 fruits each were selected and labeled on five different trees per treatment (a total of 25 branches per treatment). Each compound was sprayed six times at 10 day intervals starting at August 26 (average fruit size of 55 mm diameter). Untreated fruits on untreated trees served as controls. As disease did not appear naturally in this orchard, an artificial inoculation was performed four days following the last application in the same manner as described above. The diameter of fruit rot that developed on the skin and mesocarp of each sprayed and inoculated fruit was measured.

### 2.6. Data Analysis

Each laboratory experiment was conducted at least twice. All data were analyzed with the GLM statistical software JMP statistics package version 14.1.0 (SAS, Cary, NC, USA). For the mycelial growth on PDA or PDB, detached fruits experiments and field experiment, analysis of variance (ANOVA) was applied to colony weight, and colony or fruit rot diameters measurements. Fisher’s LSD K-ratio *t*-test was applied, to determine whether differences between treatments were significant at α = 0.05. In field experiments, treatments were arranged in a randomized complete block design.

## 3. Results

### 3.1. In Vitro Assays

#### 3.1.1. Germination

Mean conidial germination of *A. alternata* f. sp. *mali* in water was 94.7% (Table 1). Conidial germination was insensitive to BABA: percent germination in BABA of 10, 100, 1000 and 2000 μg/mL at 20 h ranged between 88.6–96.2% (Table 1). In contrast, germination in the pre-mixture azoxystrobin plus difenoconazole of 162.5 μg/mL a.i was completely inhibited.

#### 3.1.2. Mycelial Growth

**Figure 1:**
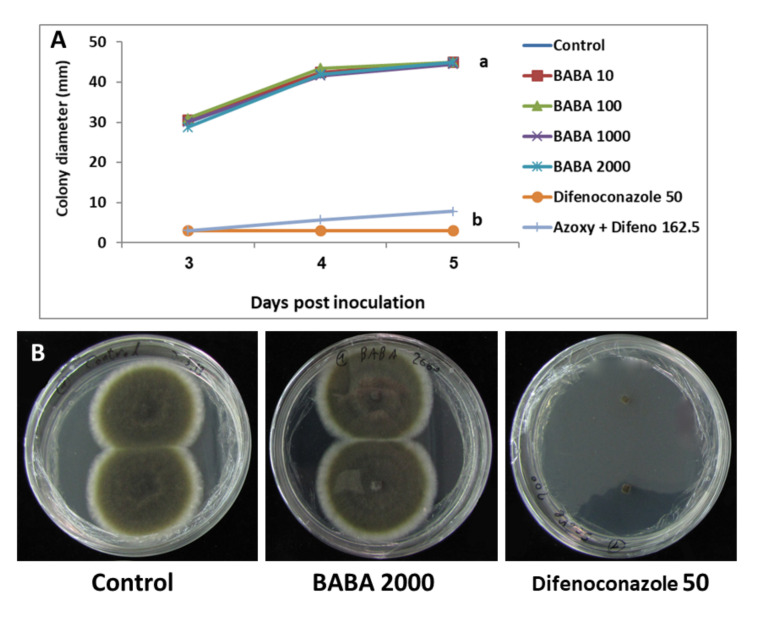
(**A**) Activity of BABA on mycelial growth of *A. alternata* f. sp. *mali* in comparison to difenoconazole (Difeno) and pre-mixture azoxystrobin plus difenoconazole (Azoxy + Difeno). Concentrations are given in μg per ml active ingredient. Different letters on curves indicate a significant (*p* < 0.05) difference between treatment (LSD test). (**B**) Activity of BABA and Score on mycelial growth of *A. alternata* f. sp. *mali* in PDA cultures. Concentrations are given in μg per mL active ingredient.

Mycelial growth of *A. alternata* f. sp. *mali* in vitro was similarly insensitive to BABA even at 2000 μg/mL (Figure 1A,B). In contrast, difenoconazole and the pre-mixture azoxystrobin plus difenoconazole provided 93% and 83% inhibition of mycelial growth, respectively, compared to water control (Figure 1A,B).

#### 3.1.3. Growth in Liquid Medium

**Figure 2:**
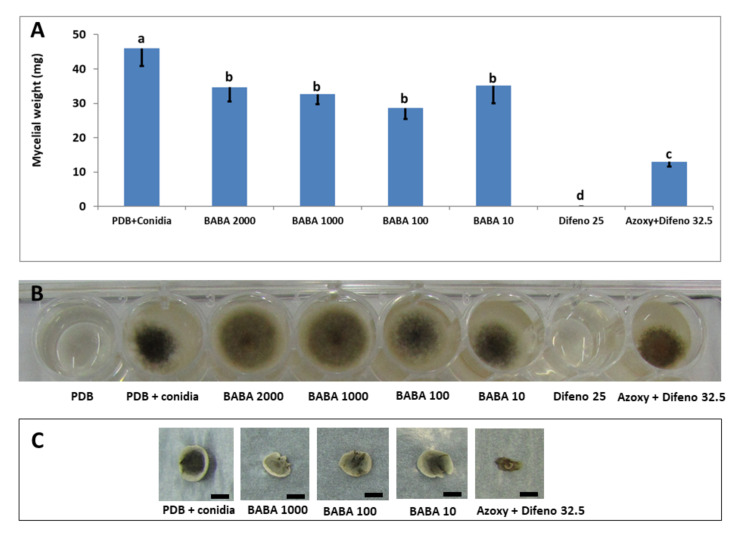
(**A**) Activity of BABA, difenoconazole (Difeno) and pre-mixture azoxystrobin plus difenoconazole (Azoxy + Difeno) on growth of *A. alternata* f. sp. *mali* in liquid medium. Concentrations are given in μg/mL of active ingredient. Bars represent the standard error of the mean. Different letters on columns indicate significant differences (*p* < 0.05) between treatments (Fisher’s LSD K-ratio *t*-test). (**B**) Representative wells of various treatments at seven days post inoculation. (**C**) Representative mycelial mats from each treatment on a blotting paper. Bar = 10 mm.

BABA of up to 2000 μg/mL had a small, though significant, inhibitory effect on fungal growth in liquid medium. The average mycelial weight of control cultures was 46 ± 5 mg, compared to 33 ± 3.5 mg in all BABA treatments (Figure 2). The pre-mixture azoxystrobin plus difenoconazole inhibited fungal growth by 72%, relative to control, while difenoconazole alone inhibited fungal growth completely (Figure 2).

### 3.2. Experiments on Detached Fruits

#### 3.2.1. Effect of Application Date on Rot Development

**Figure 3:**
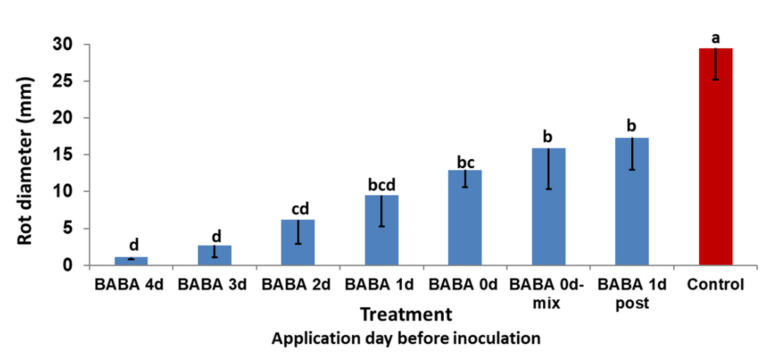
Inhibition of AFR development caused by *A. alternata* f. sp. *mali* in detached apple fruits treated with BABA at various days before or after inoculation. In treatment ‘BABA 0 d-mix’, a mixture of BABA with conidial suspension was used for inoculation. The fruit rot diameter produced at 13 days post inoculation in each inoculation site is shown. Bars represent the standard error of the mean. Different letters on columns indicate significant differences between treatments (*p* < 0.05, Fisher’s LSD K-ratio *t*-test).

Induced resistance by BABA was dependent on whether it was applied before or after inoculation. BABA was most effective in inducing resistance against *Alternaria* fruit rot when injected to the fruits at four days before inoculation (Figure 3). BABA was slightly effective even when applied 24 h post inoculation (Figure 3).

#### 3.2.2. Effect of BABA Concentration on Rot Development

**Figure 4:**
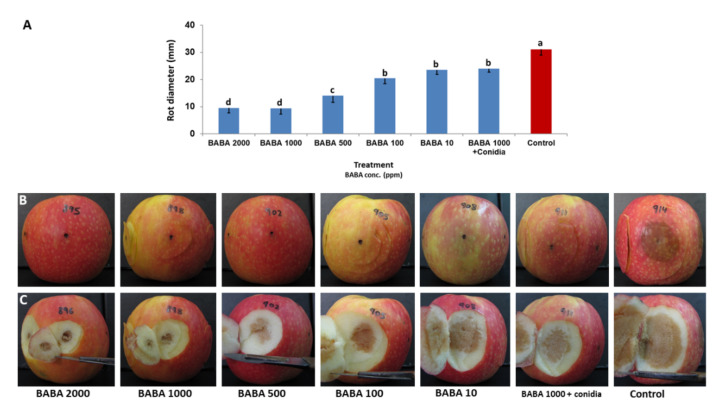
(**A**) Resistance against AFR caused by *A. alternata* f. sp. *mali* induced in detached apple fruits by BABA of various concentrations. In treatment ‘BABA 1000 + conidia’, a mixture of BABA with conidial suspension was used for inoculation. Inoculated fruits were incubated in a moist chamber at 25 °C for 13 days. The mean values represent fruit rot diameter produced in each inoculation site of each of three fruits per concentration. Bars represent the standard error of the mean. Different letters on columns indicate significant differences between treatments (*p* < 0.05, Fisher’s LSD K-ratio *t*-test). (**B**) Photos of rot developed on skin of fruits treated with BABA at various concentrations, and untreated control fruit. (**C**) Photos of rot developed in the mesoderm of fruits treated with BABA of various concentrations. Control = untreated inoculated fruit.

Fruit rot formation by *A. alternata* f. sp. *mali* was inhibited by BABA when injected into fruit four days before inoculation in a dose-dependent manner. BABA of 1000 μg/mL was the most inhibitory, providing an 82% decrease in apple blotch on fruits compared to untreated inoculated control fruits (Figure 4). A higher concentration did not improve control of rot formation.

#### 3.2.3. Efficacy of Various Isomers of BABA

**Figure 5:**
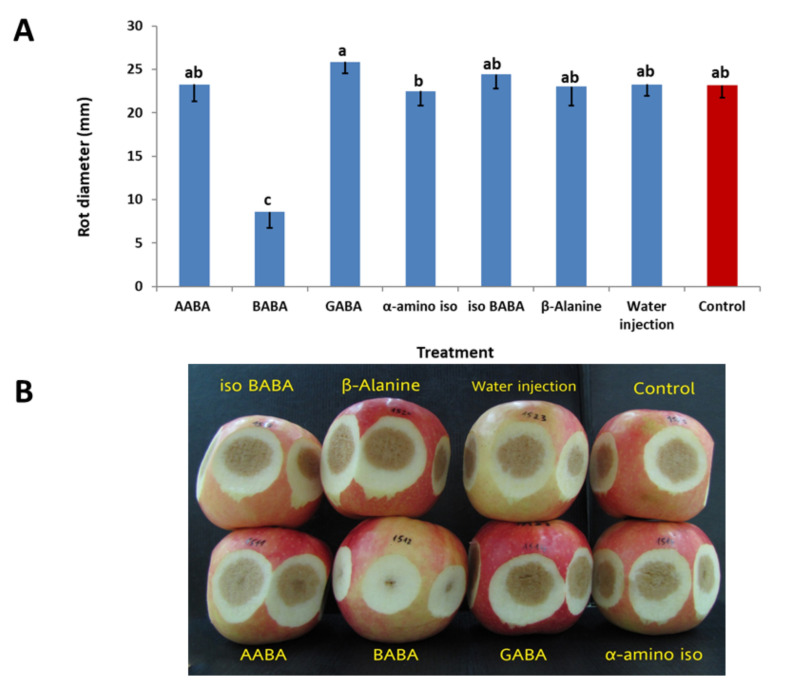
(**A**) Activity of various isomers of BABA on rot development in detached fruits. Bars represent the standard error of the mean. Different letters on columns indicate significant differences between treatments (*p* < 0.05, Fisher’s LSD K-ratio *t*-test). (**B**) Photos of rot developed in mesoderm of fruits treated with various isomers of BABA (all at 1000 ppm). Control = untreated inoculated fruit.

AABA (α-aminobutyric acid), GABA (γ-aminobutyric acid), α-amino-iso-butyric acid, dl-β-iso-BABA and 3-aminopropionic acid (β-alanine), each applied at 1000 μg/mL, were all ineffective in controlling AFR. BABA at this dose provided 63% inhibition of the symptoms relative to non-treated inoculated controls (Figure 5). Fruit rot diameter in non-treated inoculated control fruits ranged between 23 and 25 mm.

### 3.3. Field Experiments

**Figure 6:**
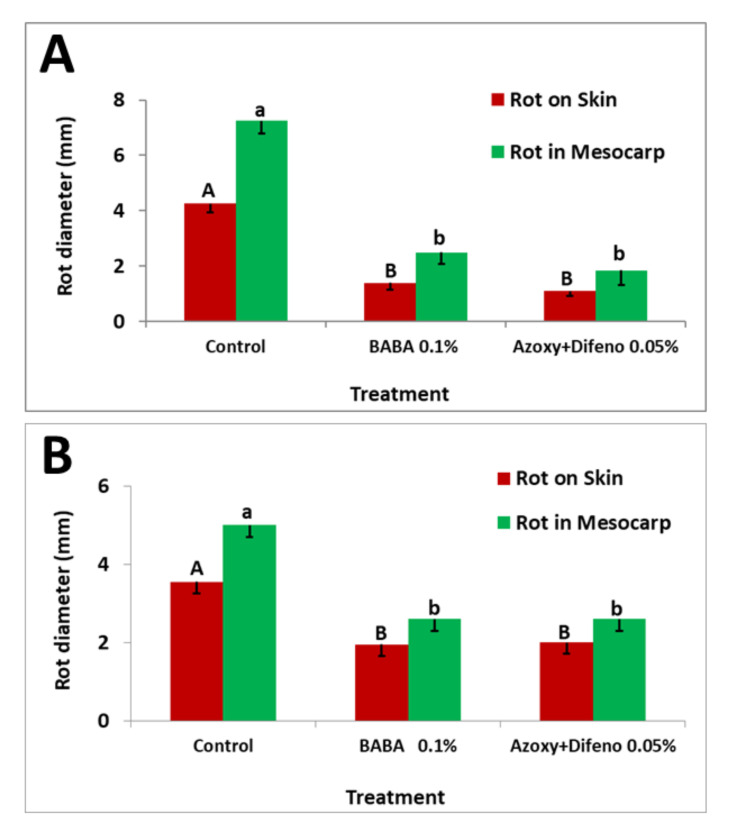
Efficacy of BABA and the pre-mixture azoxystrobin plus difenoconazole (Azoxy + Difeno) sprayed four days before inoculation on rot development in Pink Lady apple fruits in the orchard (**A**) Experiment 1. (**B**) Experiment 2. Mean values of lesion diameter on skin (uppercase letters) or in mesocarp (lowercase letters) above columns followed by different letters indicate significant (*p* < 0.05) differences between treatments (Fisher’s LSD *t*-test). Bars represent the standard error of the mean.

**Figure 7:**
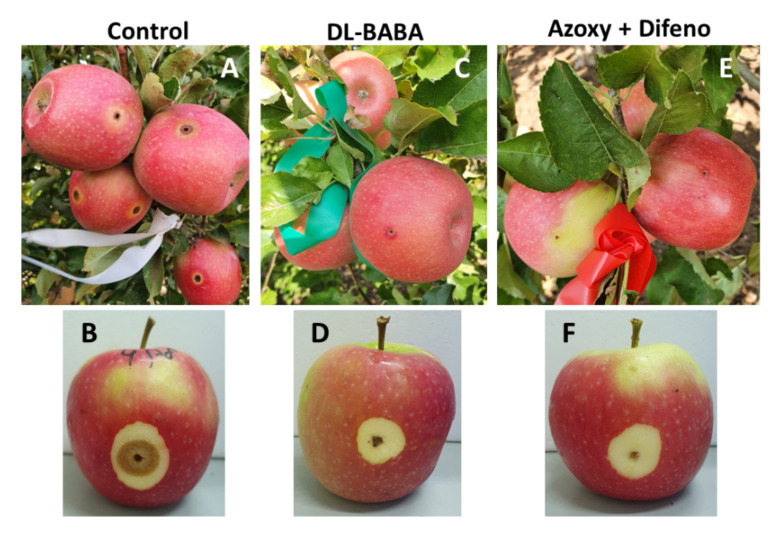
Fruit rot development following artificial inoculation in the orchard: (**A**,**B**). Rot developed on untreated fruits; (**C**,**D**) Rot developed on fruits treated with BABA; (**E**,**F**) Rot developed on fruit treated with the pre-mixture azoxystrobin plus difenoconazole (Azoxy + Difeno).

In both experiment 1 and 2, a single foliar application of BABA or of azoxystrobin plus difenoconazole applied to fruits 4 days before inoculation with *A. alternata* f. sp. *mali*, provided similar protection of the fruits, reducing fruit rot formation by 45–67% and 44–74%, respectively, compare to non-treated fruits (Figure 6A,B and Figure 7).

**Figure 8:**
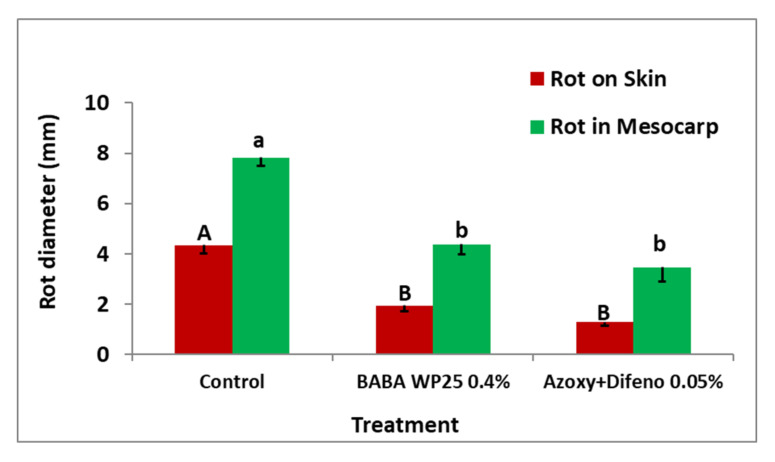
Efficacy of six foliar applications of BABA WP25 and the pre-mixture azoxystrobin plus difenoconazole (Azoxy + Difeno) on rot development in Pink Lady apple fruits in the orchard (Experiment 3). Mean values of lesion diameter on skin (uppercase letters) or in mesocarp (lowercase letters) above columns followed by different letters indicate on significant (*p* < 0.05) differences between treatments (Fisher’s LSD *t*-test). Bars represent the standard error of the mean.

In experiment 3, formulated BABA or azoxystrobin plus difenoconazole similarly reduced fruit rot formation by 55% and 70%, respectively, compare to fruits on non-treated trees (Figure 8).

## 4. Discussion

Severe outbreaks of apple fruit rot occurred in recent years in orchards of cv. Pink Lady in Israel [11,20]. Such severe fruit rot in cv. Pink Lady is not known elsewhere in the world. The causal pathogen was identified as *Alternaria alternata* f. sp. *mali* and we named the disease *Alternaria* Fruit Rot (AFR) [11]. Previous studies showed that fungicidal mixtures, such as the pre-mixture azoxystrobin plus difenoconazole, the pre-mixture captan plus tebuconazole and others, effectively controlled *Alternaria* fruit rot in apple orchards [18,20].

Plant defense activators can provide a new option for chemical management of plant diseases based on priming of host resistance [30]. There are a few reports showing resistance of fruits against fungal infection induced by plant activators [25], but no study compared their efficacy with commercial fungicides. This lack of information encouraged us to examine the activity of BABA in inducing resistance against *A. alternata* f. sp. *mali* in apple fruits. This compound was reported to induce resistance against a wide range of plant diseases [25].

We show here that BABA had no direct effect on the growth of *A. alternata* f. sp. *mali* in vitro. Spore germination and mycelial growth were insensitive to BABA (Table 1 and Figure 1). This confirms previous findings, showing that BABA had no antifungal activity in vitro [25]. In contrast, Elsherbiny et al. [31] reported that BABA of 125 mM significantly inhibited mycelial growth, spore germination and germ tube elongation of *Penicillium digitatum*. They also showed that application of BABA of 125 mM to orange fruits inoculated with *P. digitatum* suppressed disease incidence and disease severity and enhanced defense-related enzymes. Ozgonen and Karatas [32], working with *Alternaria mali*, showed that “BABA had no significant effect on mycelial growth with increasing concentration. However, the hyphal development was decreased with increasing concentration and hyphal lysis occurred just after spore germination at 800 ppm concentration”. They also showed activity of BABA against *A. mali* in leaves of young apple seedlings, but did not use apple fruits and did not show the efficacy of BABA in the field. Unlike BABA, a concentration of 162.5, 16.2 and 8 μg/mL of the pre-mixture azoxystrobin plus difenoconazole, captan and the pre-mixture captan plus tebuconazole, respectively, effectively inhibited conidial germination of *A. alternata* f. sp. *mali* (Table 1) [20].

Injection of BABA to fruits induced protection against fruit rot in a time-dependent and dose-dependent manner. The best protection was induced when BABA was applied at 1000 μg/mL (Figure 4). Injection at four days before inoculation induced the strongest protection against AFR (Figure 3). Injection of another inducer (benzothiadiazole-7-carbothioic acid S-methyl ester, BTH) provided less effective protection compared to BABA (L. Gur, *unpublished data*). BABA was also effective when applied at the same day of inoculation and even 24 h post inoculation (Figure 3). The fact that BABA was effective post inoculation indicates a rapid activation of fruit defense. Post-infection activity of BABA was reported in grape against *Plasmopara viticola* [33], tobacco against *Peronospora tabacina* [21], tomato against *Phytophthora infestans* [22] and *Bremia lactucae* in lettuce [34].

Our data corroborate with previous reports [24,25], showing that isomers of BABA (AABA, GABA, a-amino, iso BABA and β-alanine) are ineffective in inducing resistance (Figure 5). The R-enantiomer, but not the S-enantiomer, in the racemic mixture in dl-BABA, was reported to be responsible for the induced resistance against disease [24,25].

The mode of action of BABA in apple fruits against *A. alternata* f. sp. *mali* is not known. Previous studies showed that BABA induces a variety of defense mechanisms, including production of phenolics, ROS, PR proteins, callose, lignin and more, depending on the host–pathogen system [21,25,34,35,36,37]. In a recent study, Schwarzenbacher et al. [38] showed that BABA interacts with *VOZ* transcription factors to regulate abscisic acid signaling and callose-associated defense against downy mildew in Arabidopsis. The reactions of apple tissue to BABA warrants a special study.

Li et al. [39] demonstrated that BABA, BTH and 2,6-dichloroisonicotinic acid (INA), individually or in combination, were effective in suppressing progress of huanglongbing (HLB) disease caused by *Candidatus Liberibacter asiaticus* in citrus trees in the field. These treatments also conferred a positive effect on fruit yield and quality. They concluded that plant defense inducers may be a useful strategy for management of citrus HLB disease.

The present research shows that BABA induced an effective protection of apple fruits against *A. alternata* f. sp. *mali* in either artificially inoculated detached apples in the laboratory or following artificial fruit inoculations in the orchard. A single foliar application of either BABA or azoxystrobin plus difenoconazole to field-grown fruits reduced fruit rot by 45–67% and 44–74%, respectively, compared with non-treated control shoots (Figure 6A,B and Figure 7). A previous study showed that fruits of cv. Pink Lady acquired susceptibility to the disease at about 115 days after petal fall (DAPF) when reaching a diameter of ≥55 mm about 15 days before the appearance of fruit cracking [18]. On this basis, another experiment involving six foliar sprays of either BABA or azoxystrobin plus difenoconazole were applied to field-grown fruits. As disease did not show up, an artificial inoculation was carried out four days after the last foliar spray. Again, early applications of BABA induced protection against *A. alternata* f. sp. *mali* and were as effective as the commercial fungicidal mixture azoxystrobin plus difenoconazole in reducing the formation of fruit rot (Figure 8). A similar controlling effect of BABA was observed against moldy core disease (*Alternaria alternata*) in apple orchards [28].

These findings may encourage the use of BABA as an alternative to the fungicidal mixture azoxystrobin plus difenoconazole for the control of *Alternaria* fruit rot. BABA has no direct antifungal activity and therefore poses no risk for developing fungal resistance. If incorporated in IPM programs, BABA can prevent the build-up of resistance against commercial fungicides. Reduced sensitivity of *A. alternata* f. sp. *mali* to polyoxin and pyraclostrobin was already reported in apple [40,41,42]. BABA may be alternated or combined with products such as triazoles or strobilurins, towards which the pathogen has shown reduced sensitivity, and thus extend their useful life [43].

This is the first report on the capacity of BABA to induce resistance against *A. alternata* f. sp. *mali* in detached apple fruits and *Alternaria* fruit rot in the field. The control of *Alternaria* fruit rot with a plant activator such as BABA may become a component of integrated disease management, enabling control of the disease with reduced application of fungicides.

## Figures and Tables

**Table 1:** Activity of BABA and the pre-mixture azoxystrobin plus difenoconazole on *A. alternata* f. sp. *mali* conidia germination.

Treatment (Conc. µg/mL)	% Germinated Conidia	SE ^2^
Control	94.7	2.80
BABA 10	93.7	3.33
BABA 100	92.5	2.75
BABA 1000	95.4	2.46
BABA 2000	88.6	3.16
Azoxy + Difeno 162.5 ^1^	0 *	0

^1^ Concentration given in µg/mL a.i of the prepacked mixture of azoxystrobin (200 g/L) + difenoconazole (125 g/L). ^2^ SE = standard error. * Significantly different (*p* < 0.05, Fisher’s LSD K-ratio *t*-test).

## Data Availability

This study did not report any data.
